# Dynamics of Lamin-A Processing Following Precursor Accumulation

**DOI:** 10.1371/journal.pone.0010874

**Published:** 2010-05-28

**Authors:** Qian Liu, Dae In Kim, Janet Syme, Phyllis LuValle, Brian Burke, Kyle J. Roux

**Affiliations:** 1 Department of Histology and Embryology, Shandong University School of Medicine, Jinan, Shandong, China; 2 Department of Anatomy and Cell Biology, University of Florida, Gainesville, Florida, United States of America; 3 Institute of Medical Biology, Immunos, Singapore, Singapore; Institut Pasteur Korea, Republic of Korea

## Abstract

Lamin A (LaA) is a component of the nuclear lamina, an intermediate filament meshwork that underlies the inner nuclear membrane (INM) of the nuclear envelope (NE). Newly synthesized prelamin A (PreA) undergoes extensive processing involving C-terminal farnesylation followed by proteolysis yielding non-farnesylated mature lamin A. Different inhibitors of these processing events are currently used therapeutically. Hutchinson-Gilford Progeria Syndrome (HGPS) is most commonly caused by mutations leading to an accumulation of a farnesylated LaA isoform, prompting a clinical trial using farnesyltransferase inhibitors (FTI) to reduce this modification. At therapeutic levels, HIV protease inhibitors (PI) can unexpectedly inhibit the final processing step in PreA maturation. We have examined the dynamics of LaA processing and associated cellular effects during PI or FTI treatment and following inhibitor washout. While PI reversibility was rapid, with respect to both LaA maturation and associated cellular phenotype, recovery from FTI treatment was more gradual. FTI reversibility is influenced by both cell type and rate of proliferation. These results suggest a less static lamin network than has previously been observed.

## Introduction

The nuclear lamina is an intermediate filament meshwork composed of A- and B-type lamins. In mammalian somatic cells the A-type lamins are represented by lamins A and C (LaA/C), which arise through alternative splicing of the *LMNA* gene. Several diseases are associated with mutations in *LMNA*, including progeria, lipodystrophy, muscular dystrophy and peripheral neuropathy [1]. LaA and LaC differ only by virtue of unique C-terminal extensions, 96 residues for LaA and six for LaC [2]. Unlike LaC, LaA undergoes multistep posttranslational processing at its C-terminal CaaX motif involving 3–4 enzymes. Initially a farnesyl moiety is added to the cysteine by farnesyltransferase, followed by cleavage of the last three amino acids (-aaXing) by either Rce1 or ZmpSte24. This C-terminal cysteine is carboxymethylated by isoprenylcysteine carboxymethyl transferase (ICMT) to generate farnesylated/carboxymethylated-PreA. Within approximately 90 min of synthesis [3], ZmpSte24 cleaves PreA [4], [5], [6] 14 amino acids upstream of the C-terminus to generate mature LaA [7], [8]. PreA is the only known substrate of ZmpSte24 [9].

Defective LaA processing has been implicated in both familial and acquired forms of lipodystrophy. Mutations in *LMNA* that alter certain charged residues on the surface of the Ig-fold region common to both LaA and LaC are associated with Dunnigan-type familial partial lipodystrophy (FPLD) [10], [11]. FPLD patients exhibit peri-pubertal onset of subcutaneous fat loss from the extremities and trunk, hypercholesterolemia and type-II diabetes [12], [13]. There are reports of PreA accumulation in FPLD-patient fibroblasts through an unknown mechanism [14], [15]. PreA accumulation is also observed in cells from HIV-infected patients with acquired lipodystrophy [15], [16]. This is likely related to certain HIV PIs used in highly active antiretroviral therapy (HAART) that inhibit the activity of ZmpSte24 [17], [18], [19]. Conclusive evidence for an involvement of PreA in HAART-associated lipodystrophy has yet to be presented.

Incomplete LaA processing is also associated with the rare premature aging disorder, HGPS, in which patients begin to exhibit a phenocopy of premature ageing around 1–2 years and die of cardiovascular-related illness by around 13 years of age [20], [21], [22]. The most common HGPS mutation (LaA G608G) generates a cryptic splice site within exon 11 of *LMNA* resulting in deletion of 50 amino acid residues within the LaA C-terminus. This truncated LaA, termed progerin, lacks the second cleavage site for ZmpSte24, resulting in retention of the farnesylated and carboxymethylated C-terminal cysteine [23], [24], [25]. In support of farnesylated-PreA or -progerin toxicity [9], [26] is restrictive dermopathy (RD), a perinatal lethal disease with progeroid features in which farnesylated PreA accumulates due to mutations in *ZMPSTE24* or *LMNA* [26], [27]. Although the mechanism of progerin or farnesylated-PreA toxicity remains unclear, these observations led to studies that tested the efficacy of FTI in ameliorating HGPS symptoms by inhibiting farnesylation of LaA along with the other CaaX-motif proteins (there are ∼100 predicted in the human genome). In cell culture and mouse progeria models, FTIs yielded promising results with improved nuclear morphology *in vitro* [28], [29], [30], [31], [32], and weight gain with increased viability *in vivo* [33], [34], [35]. Thus, a clinical trial for HGPS patients with the FTI, lonafarnib, was rapidly established, the outcome of which remains unreported [36].

Despite the clinical use of the LaA processing inhibitors, FTIs and HIV PIs, there are fundamental deficits in our understanding of the consequences. After prolonged treatment with either form of inhibitor, the nuclear lamina will contain considerable levels of PreA, either farnesylated or non-farnesylated. Would rapid processing of this PreA occur following inhibitor release, or would lamina disassembly during cell division be required? This question has implications in terms of the reversibility of both FTIs and PIs, which may in turn impact their clinical usage. Such reversibility, or lack thereof, would also shed new light on lamina dynamics and interactions within the nuclear lamina. In this study, we have explored the recovery rates for LaA processing following accumulation of both farnesylated and non-farnesylated forms of PreA within the nuclear lamina.

## Methods

### Reagents and Treatment

HIV protease inhibitors were obtained from the National Institutes of Health AIDS Research and Reference Reagent Program. Nelfinavir, lopinavir and atazanavir sulfate were prepared at a stock solution at 20mM in DMSO, indinavir sulfate at 20mM in water, tipranavir at 20mM in ethyl acetate. FTI-277 (Sigma) was prepared as a stock at 10mM. Lov (Sigma, St. Louis, MO) was used at 10µM and GGTI-2147 (Calbiochem) at 20µM. BMS-214662 was a gift from M. Gelb, University of Washington. Unless otherwise noted, Saos-2 cells were treated for 48 hr at 20µM for HIV PIs, Lov and GGTI-2147, and at 10µM for FTI-277. Cycloheximide was used at 10µg/ml. Mitomycin C was used at 10 µg/ml (2 hrs at 37°C). Control cells were incubated with the vehicle DMSO or ethyl acetate. HGPS fibroblasts were treated with FTI-277 at a dose of 10µM for 4–9 days.

### Cell Culture

Saos-2 cells were maintained in 6% CO2 at 37°C in DMEM (GIBCO BRL) with 10% FBS (Hyclone), and 10% penicillin/streptomycin (GIBCO BRL). Human G608G HGPS fibroblasts were obtained from the Coriell Cell Repository (repository nos. AG01972) and maintained in 6% CO2 at 37°C in DMEM with 15% FBS, 10% penicillin/streptomycin and 2× concentration of essential and non-essential amino acids. To washout the inhibitors, cells were washed with PBS times and culture media three times. Cells were transfected as described previously [37].

### Antibodies

The following antibodies were used in this study: mouse anti-LaA/C [38], anti-Nup153 [39], anti-HDJ-2 (MS-225, Thermo Scientific); rabbit anti-Sun-2 [40], anti-nesprin-3 (gift from A. Sonnenberg, Netherlands Cancer Institute, Amsterdam, NL), anti-emerin (gift from G. Morris, Robert Jones and Agnes Hunt Orthopaedic Hospital, Oswestry, UK), anti-LaA/C (Cell Signaling), anti-LaB1 (ab16048, Abcam), anti-LaA (SC20680) and goat anti-LaA (SC6214), anti-LaA/C (SC621) (Santa Cruz Biotechnology). Secondary antibodies conjugated with AlexaFluor dyes (Invitrogen) or peroxidase (Biosource International) were used as previously described [37].

### Immunofluorescence Microscopy

For immunofluorescence microscopy, cells were grown on glass coverslips and fixed, permeabilized, immunolabeled and observed as described previously [37]. Image quantification was performed using IPLab software and NIH ImageJ Circularity software. Unpaired t-tests were performed to evaluate the significance of the results.

### Immunoblotting-pulse chase immunoprecipitation

Subconfluent 35-mm dishes of Saos-2 cells, either untreated or treated with DMSO, Lop, Lov, or FTI-277 for 48 hrs, were incubated in 90% DMEM with 10% FBS, and 10% L-cysteine and L-methionine-free media (MP Biolabs) with 25 µCi ^35^S Translabel (MP Biolabs). After 18 hrs, Saos-2 cells were washed once with PBS and refed with DMEM plus 10% FBS with or without inhibitors. One hr later, Saos-2 cells were washed twice with PBS and either incubated in DMEM with 10% FBS for an additional 1–24 hr or lysed immediately. For cell lysis, cells were washed three times in PBS and incubated in 800µl lysis buffer (50 mM Tris-HCl, pH 9.0; 500 mM NaCl, 0.4% SDS, 2% TX-100, 1mM dithiothreitol 10mg/ml in DMSO each of chymostatin, leupeptin, antipain, and pepstatin) on ice for 5 min. The cells were then scraped off and sheared 8× through a 26-gauge needle. After centrifugation for 10 min at 10,000×G, the supernatant was incubated overnight at 4°C with rabbit anti-LaA/C and protein A-sepharose beads. Beads were washed three times in lysis buffer and once in wash buffer (50 mM Tris-HCl, pH 7.4; 50 mM NaCl). Samples were processed for SDS-PAGE and fluorography [41]. All Densitometry was performed using NIH ImageJ.

## Results

**Figure 1:**
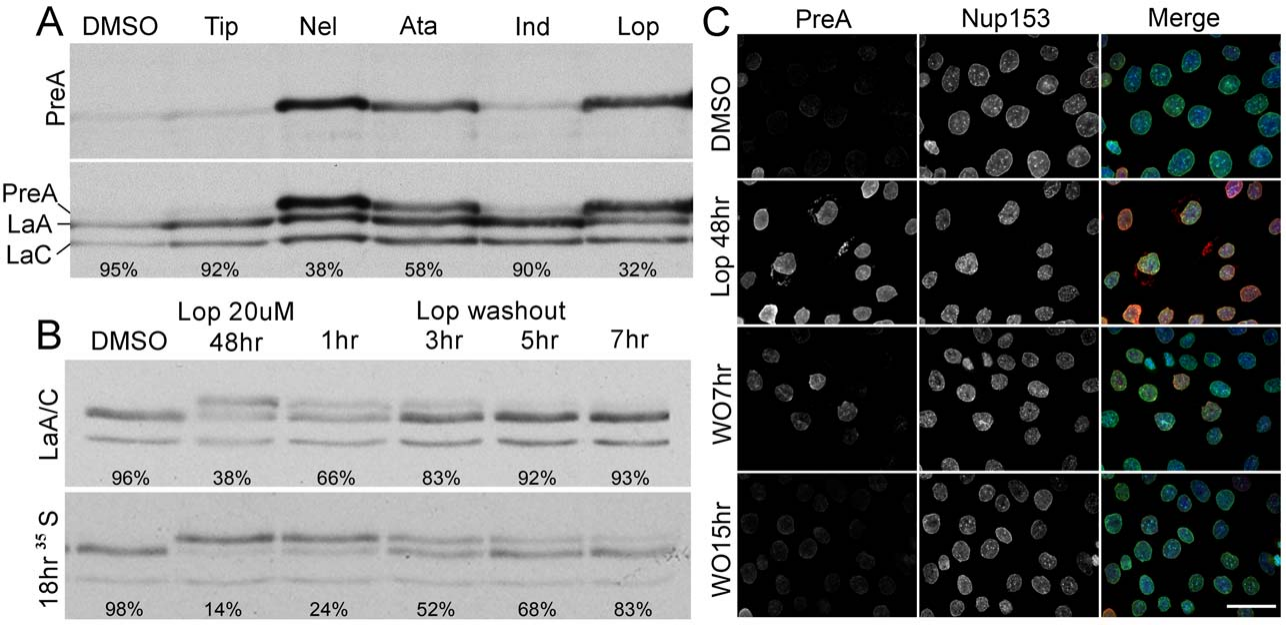
HIV PIs block LaA maturation, which is reversed rapidly following PI washout. (A) Blotted with PreA specific and LaA/C antibodies, extracts from Saos-2 cells treated with vehicle (DMSO), Tip, Nel, Ata, Ind and Lop reveal different levels of PreA accumulation. Percentage of mature LaA is listed under each lane. (B) Immunoblot of extracts from Saos-2 cells treated with Lop followed by washout indicates that half time of LaA maturation is approximately 3 hrs. The processing rate of the overnight ^35^S Cys/Met labeled LaA following Lop washout is similar. Percentage of mature LaA is listed under each lane. (C) Immunofluorescence microscopy of Saos-2 cells double labeled with the antibodies again PreA and Nup153. PreA is accumulated on the NE after Lop treatment and disappears following Lop washout. All images are taken at the same exposure time. In the merged images, DNA, revealed by staining with Hoechst dye, is shown in blue. Bar, 15µm.

To explore PreA processing following treatment of human cells with HIV protease inhibitors, we first examined their efficacy in promoting farnesylated PreA accumulation. Saos-2 cells were treated for 48h with various HIV-PIs and the relative levels of PreA determined by immunoblot analysis. Lopinavir (Lop) and nelfinavir were most effective in blocking the PreA maturation, followed by atazanavir; whereas indinavir and tipranavir had little effect on PreA accumulation (Figure 1A). As Lop provided the best balance between cellular toxicity and PreA accumulation, we used it exclusively for the remainder of our studies.

In order to determine the susceptibility of PreA accumulated at the NE to proteolytic maturation, Saos-2 cells were treated for 48 hrs with Lop, prior to washout of the drug. Before drug withdrawal, more than half of the total cellular LaA was detected as PreA (Figure 1B, upper panel). However, by 3 hrs after Lop washout ∼50% of PreA was processed to maturity and by 5–7 hrs the levels approached that of the control. Parallel immunofluorescence experiments reveal the accumulation of PreA at the NE (Figure 1C). Loss of PreA following Lop washout follows a time-course similar to that observed by western blot. Complementary pulse-chase experiments were performed on Saos-2 cells labeled overnight with ^35^S Cys/Met in the presence of Lop (Figure 1B, lower panel). After labeling, the cells were cells were “chased” for 1h in medium containing both Lop and excess non-radioactive Cys/Met to ensure newly synthesized labeled lamins were incorporated into the nuclear lamina. The chase was continued for 7h in the absence of Lop. Roughly 50% of the accumulated PreA is processed to the mature form by 3h, and by 7h it is fully processed (Figure 1B, lower panel). To confirm that our results do not reflect turnover of accumulated PreA in combination with *de novo* synthesis, protein synthesis was inhibited with cyclohexamide 1 hr prior to and during the Lop washout (Figure S1). The rate of PreA processing appears enhanced in the presence of cycloheximide, most likely due to the elimination of any newly synthesized PreA as a competitive substrate for ZmpSte24. In normal tissue culture cells, the half-time for newly synthesized PreA processing is ∼1.5 hrs [3]. It is remarkable that the recovery half-time from extended Lop treatment is only ∼3h, especially as the bulk of the total PreA in the Lop-treated cells appears integrated into the nuclear lamina.

**Figure 2:**
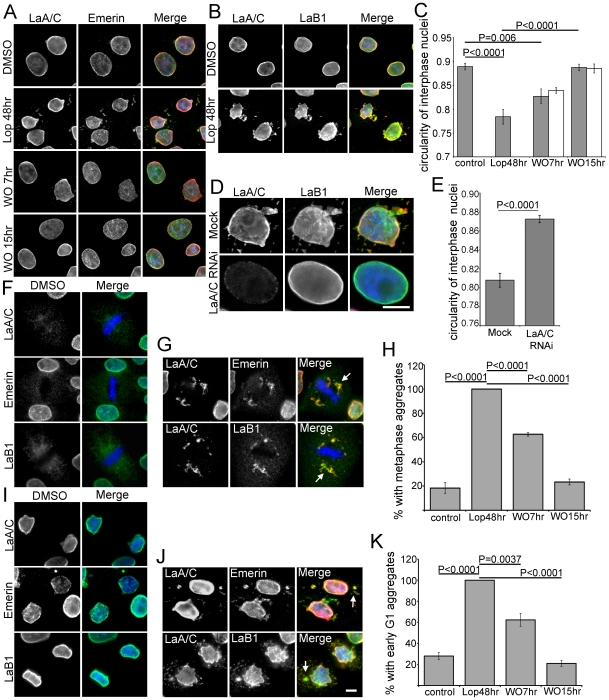
Recovery from aberrant cellular phenotypes is delayed following Lop washout. (A) Immunofluorescence microscopy of Saos-2 cells. Altered interphase nuclear morphology and abnormal accumulation of LaA/C and emerin in the cytoplasm are evident after Lop treatment and recover within 15 hrs following Lop washout. (B) The aberrant cytoplasmic aggregates after Lop treatment contain LaB1, predominantly colocalized with LaA/C. (C) Nuclear circularity following Lop washout is significantly altered by PI treatment and following washout either without (grey bars) or with cycloheximide (white bars) (error bars = SEM; N = >96). (D) Double labeled with antibodies against LaA/C and LaB1, Saos-2 cells exhibit normal nuclear morphology after Lop treatment following LaA/C RNAi. (E) Nuclear circularity is higher in LaA/C RNAi treated cells (error bars = SEM; N = >65). (F) Nuclear components such as LaA/C, emerin and LaB1 are diffuse in control cells during metaphase. (G) Lop treatment leads to aberrant aggregation of LaA/C adjacent to metaphase chromosomes. Emerin and LaB1 are also retained in these aggregates (white arrows). (H) Measurements of percentage of cells with metaphase aggregates indicate that mitotic abnormality recovers by ∼15 hrs after Lop washout (error bars = SEM; N = 3, >75 cells counted each experiment). (I) LaA/C, emerin and LaB1 localize on the NE in early G1. (J) Lop treatment leads to aberrant aggregation of LaA/C in the cytoplasm in early G1. These aggregates also contain emerin and LaB1 (white arrows). (K) Measurements of percentage of cells with early G1 aggregates indicate that recovery of cytoplasmic abnormality is ∼15 hrs after Lop washout (error bars = SEM; N = 3, >75 cells counted each experiment). Bars, 5µm.

Following extended treatment with PIs, and concomitant with PreA accumulation, cells acquire irregular nuclear profiles with quantifiably decreased circularity (Figure 2A–C). Normal nuclear morphology recovers independent of new protein synthesis over a period of 7–15h following Lop washout (Figure 2C). To determine if the effect of Lop on nuclear morphology is LaA-dependent Saos-2 cells were depleted of LaA and LaC by RNAi in conjunction with Lop treatment (Figure 2D–E). Cells depleted of LaA and LaC retained normal nuclear morphology and circularity over the 48h period of the experiment suggesting that PreA is the mediator of Lop-induced nuclear dysmorphology. Another consequence of HIV PI-treatment is the aberrant accumulation of LaA and LaC in cytoplasmic aggregates in both mitotic and early G1 cells (Figure 2F–K). In the former, aggregates tend to be adjacent to the spindle poles. Similar aggregates have been described in cells expressing progerin [42], [43]. We also observed a range of other NE proteins, including emerin, LaB1 (Figure 2G, J), sun-2 and nesprin-3 (Figure S2) that were also retained within both the mitotic and G1 aggregates. After Lop washout the frequency of these aggregates diminished to control levels over a period of 7–15h, paralleling the restoration of normal nuclear morphology (Figure 2H, K).

**Figure 3:**
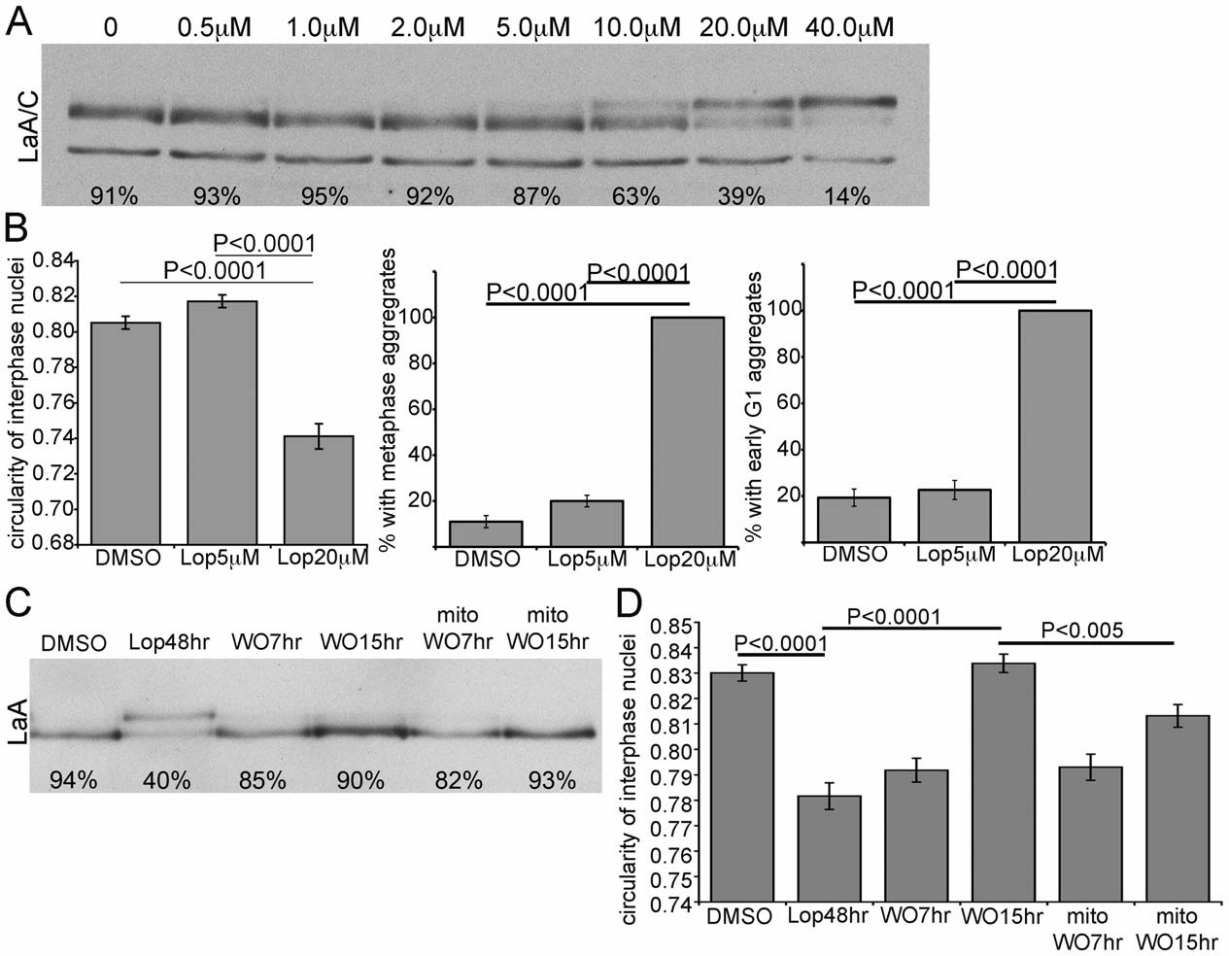
Recovery of nuclear morphology following Lop washout is time dependent and enhanced in cycling cells. (A) Probed with LaA/C antibody, immunoblot of Saos-2 cells treated with increased concentrations of Lop from 0 to 40 µM reveals increased PreA accumulation in parallel. Percentage of mature LaA is listed under each lane. (B) Measurements of interphase nuclear circularity (error bars = SEM; N = >80), percentage of cells with metaphase and early G1 aggregates (error bars = SEM; N = 3, >75 cells counted each experiment) reveal that the effects of 5µM Lop differs significantly from 20µM Lop, with similar level to vehicle (DMSO) control. (C) Accumulated PreA in Lop treated cells complete its maturation by 7 hrs following drug washout. Mitomycin C treatment prior to Lop removal does not affect PreA processing rate. Percentage of mature LaA is listed under each lane. (D) Measurement of nuclear circularity indicates that mitomycin delays recovery of aberrant nuclear shape at 15 hrs following Lop washout (error bars = SEM; N = >92).

Considering the half-time of PreA maturation is ∼3hrs after Lop washout, what is the cause of this delay in morphological recovery? One explanation is that nuclear and/or nuclear envelope remodeling must take place during the lag period. Another possibility is that the level of farnesylated-PreA at the time of washout is far in excess of that required to induce these nuclear abnormalities. To attempt to resolve this issue, we first determined the lowest concentration of Lop that after 48h would produce an accumulation of farnesylated-PreA equivalent to that observed 5–7h after washout of our normal 20µm Lop (Figure 3). The concentration of Lop chosen was 5µm. Subsequently we compared nuclear circularity and appearance of LaA/C aggregates during metaphase and early G1 from cells treated with 5µM or 20µM Lop (Figure 3B). 5µM Lop was unable to induce a significant change in nuclear morphology or to induce LaA/C aggregation. These data imply that nuclear or nuclear envelope remodeling likely accounts for the lag period, although a threshold for PreA accumulation causing nuclear dysmorphology cannot be entirely discounted. We next determined whether such remodeling might require cell division. Cells were treated with mitomycin C prior to Lop washout to prevent cell division. In treated cells, we observed no difference in the maturation rate of farnesylated PreA (Figure 3C). However, we did observe a significant delay in recovery of nuclear circularity (Figure 3D). The suggestion is that postmitotic nuclear reassembly partially contributes to the restoration of normal nuclear morphology. However, we consider it likely that ongoing synthesis of NE components and their incorporation into interphase nuclei may also contribute to recovery from Lop treatment.

**Figure 4:**
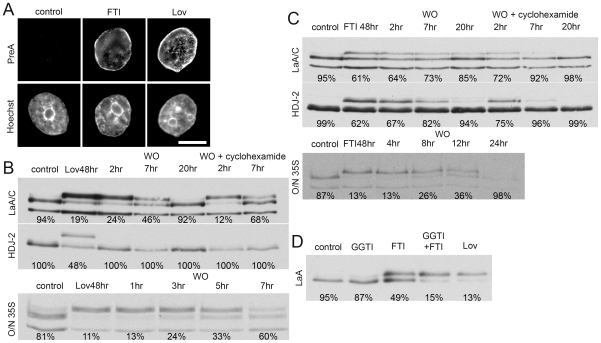
Nonfarnesylated-PreA accumulated at the NE by prenylation inhibitors gradually matures following drug washout. (A) Immunofluorescence microscopy of Saos-2 cells reveals significant accumulation of nonfarnesylated-PreA on the NE after a treatment of Lov or FTI-277. DNA, revealed by staining with Hoechst dye, is shown at bottom column. Bar, 5µm (B) Immunoblot of extracts from Saos-2 cells treated with Lov followed by washout indicates that half-time of PreA maturation is ∼7 hrs. Cyclohexamide chase upon release from Lov block enhances maturation. Accumulations of slower migrating non-farnesylated HDJ-2 regain farnesylation by 2 hrs following Lov washout. Processing rate of overnight ^35^S Cys/Met labeled LaA following Lov removal is similar to unlabeled total population. (C) In similar experiments with FTI-277, the half-time of PreA maturation following FTI-277 washout is ∼15 hrs in total lysates, ∼7 hrs with cyclohexamide chase and ∼12 hrs in an overnight ^35^S Cys/Met labeled population. HDJ-2 is farnesylated at a faster rate than LaA is processed upon FTI-277 washout. (D) Immunoblot of extracts of Saos-2 cells probed with LaA antibody. In contrast to the incomplete PreA accumulation by FTI-277 treatment, a combinatorial treatment of FTI-277 and GGTI-2147 or Lov-alone lead to more complete accumulation of non-prenylated PreA. Percentage of mature LaA or HDJ-2 is listed under each lane.

Our next question was whether an accumulation of lamina-associated non-farnesylated PreA, such as following long-term FTI treatment, would be processed to maturity with similar efficiency to that observed for farnesylated-PreA. We first examined the efficacy of both lovastatin (Lov) and the farnesyl transferase inhibitor FTI-277 in accumulating non-farnesylated PreA in Saos-2 cells. Lov is an inhibitor of the enzyme HMG-CoA reductase which catalyses the production of mevalonic acid, a key precursor of isoprenoid synthesis. Treatment with Lov will consequently block prenylation involving both farnesyl (C15) and geranylgeranyl (C20) modifications. Both inhibitors induced accumulation of non-farnesylated PreA at the NE of Saos-2 cells treated for 48 hrs (Figure 4A). Following Lov washout the half-time of mature LaA recovery was ∼7h as determined by both western blot and pulse-chase analysis (Figure 4B). Similar studies with FTI-277, employing both western blot and pulse-chase analyses, revealed a considerably slower restoration of non-farnesylated PreA processing with a recovery half-time of ∼12h (Figure 4C). With either treatment, inhibition of new protein synthesis with cyclohexamide added at drug washout accelerated PreA maturation, likely due to reduced competition from newly synthesized substrates. Analysis of the processing of HDJ-2, another farnesylated protein, reveals that washout of lovastatin is rapid whereas the processing of LaA is delayed (Figure 4B). However, in the case of FTI washout, recovery of HDJ-2 farnesylation is slowed, but not as markedly so as the maturation of non-farnesylated pre-LaA (Figure 4C). We repeated the same experiments with other FTIs such as L-744, 832 and BMS-214662 and observed the same effect (Figure S3).

**Figure 5:**
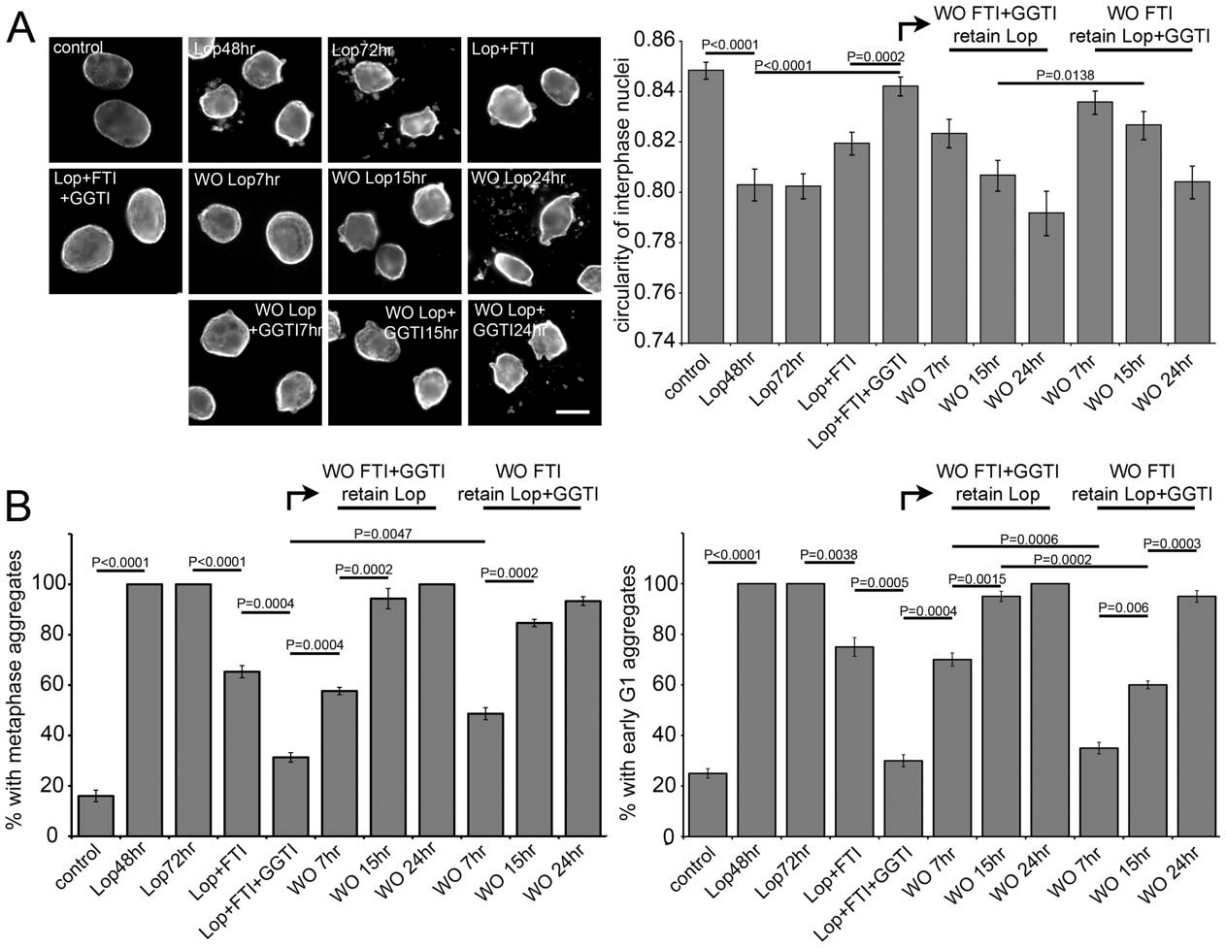
Aberrant cellular phenotypes recover rapidly in PI treated cells following washout of FTI and GGTI. (A) Labeled with LaA/C antibody, immunofluorescence microscopy of Saos-2 cells reveals that the combination of FTI-277 and GGTI-2147, but not FTI-alone, rectify aberrant nuclear morphology and abnormal nucleoplasmic aggregation caused by Lop. Release of FTI and GGTI enhance this phenotypic rebound. Bar 10µm. (B) Measurements of nuclear circularity and percentage of cells with metaphase and early G1 aggregates reveal that corresponding cellular phenotypes are reversed by 15 hrs following FTI-277 and GGTI-2147 washout and by 24 hrs following FTI-277 washout (error bars = SEM; N = 3, >75 cells counted each experiment).

Both Lov and FTIs led to significant accumulation of the slower migrating non-farnesylated-PreA, however lovastatin was considerably more effective than any of the FTIs. Recently it was shown that in the absence of farnesylation, PreA could be a substrate for geranylgeranylation [44]. To explore this possibility, Saos-2 cells were treated with geranylgeranyl transferase inhibitor (GGTI)-2147 alone or in combination with FTI (Figure 4D). While GGTI-alone had no effect on LaA maturation, and the FTI alone had only a partial effect, in combination the GGTI and FTI largely blocked LaA processing, yielding results similar to those obtained with lovastatin. These results support the concept that farnesyltransferase inhibition induces geranylgeranlyation and maturation of a substantial fraction of LaA. If the cytotoxic affects of Lop, including nuclear dysmorphology and the appearance of mitotic and G1 LaA/C-containing aggregates, can be ascribed to the accumulation of farnesylated PreA, then inhibition of farnesyltransferase should abrogate these effects. To test this idea, Saos-2 cells were treated with Lop, both with and without FTI, for periods of up to 72h (Figure 5A–B). Lop alone, as expected, caused nuclear deformation (measured as loss of circularity) and mitotic and G1 lamin-positive aggregates. Inclusion of FTI partially suppressed these effects; whereas FTI and GGTI together almost eliminated Lop-induced nuclear dismorphology and cytoplasmic lamin aggregates. Subsequent washout of both FTI and GGTI together or of FTI alone (leaving GGTI in the medium) resulted in the re-appearance of Lop cytotoxicity over a period of about 24h. Reappearance of dysmorphology was more rapid when both FTI and GGTI were washed out together. The implication is that non-prenylated PreA that has been accumulated over an extended period can be utilized as a substrate by geranylgeranyl transferase even in the presence of active farnesyl transferase.

**Figure 6:**
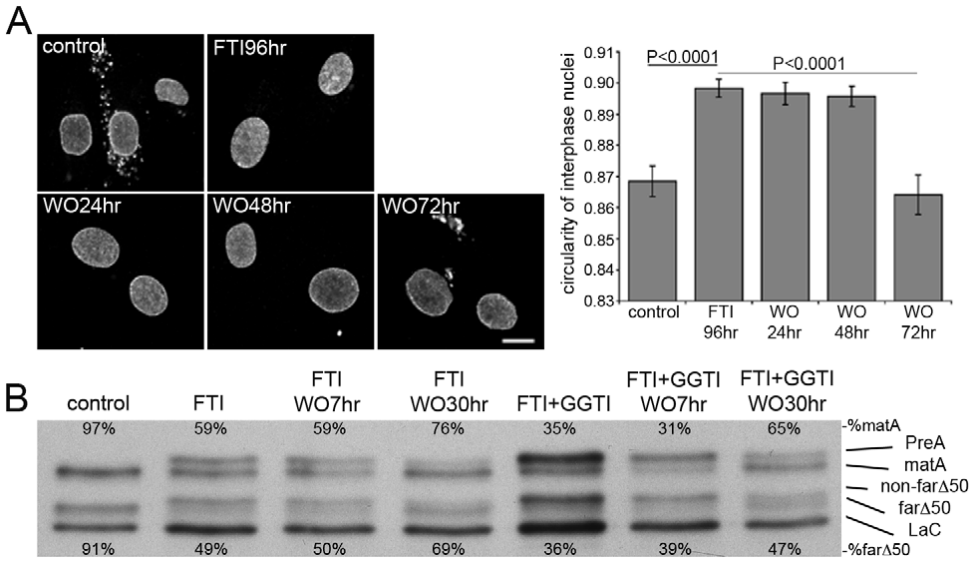
LaA and progerin are slow to mature in HGPS cells following FTI washout. (A) FTI-277 treatment of HGPS cells for 96 hrs inhibits abnormal nuclear morphology as observed by LaA/C imunofluorescence (error bars = SEM; N = >80). Bar, 10µm. (B) After a 9 day FTI-277 treatment, ∼50% of the LaA is PreA and nonfarnesylated-progerin (non-farΔ50). In contrast FTI-277 and GGTI-2147 lead to a more complete accumulation of PreA and non-farΔ50. Following FTI or FTI and GGTI washout, mature LaA (matA) and farnesylated progerin (farΔ50) slowly recover, but at relatively similar rates. Percentage of mature LaA or LaAΔ50 is listed above or below each lane, respectively.

That FTI treatment permits geranylgeranylation of LaA is of particular significance given that FTI treatment of HGPS patients is currently the subject of a clinical trial. The effectiveness of this treatment strategy could be compromised by alternative lamin prenylation. To begin to address this issue we first established that FTI treatment indeed restored normal nuclear morphology, as previously reported [28], [29], [30], [31], [32], and eliminated cytoplasmic lamin-positive aggregates in HGPS dermal fibroblasts (Figure 6A). Upon washout of the FTI, it took on the order of 48–72h to reacquire the aberrant HGPS morphology. Western blot analysis of cells after FTI treatment reveals a partial shift to slower migrating forms for both progerin and LaA. While progerin does not undergo ZmpSte24-mediated maturation, it does display a mobility shift linked to prenylation and -aaXing (Figure 6B, S4A). FTI washout revealed slow processing of both non-farnesylated PreA and non-farnesylated progerin over a period of about 30h, significantly longer than that observed in Saos-2 cells. Similarly, HDJ-2 required a longer period to mature in HGPS cells (Figure S4B). Combined treatment of HGPS fibroblasts with both FTI and GGTI resulted in a more substantial shift of LaA to PreA (Figure 6B). Albeit less dramatic, an increase in the accumulation of non-farnesylated progerin was also observed. Thus our data support the findings that in the absence of farnesylation, both full-length PreA and, to a lesser extent, progerin can be geranylgeranylated in HGPS dermal fibroblasts [44]. However, washout of both FTI and GGTI did not appreciably enhance the rate of return to the mature forms.

## Discussion

In this study we have evaluated the processability of PreA localized to the NE following the use of HIV PIs or FTIs. Significant levels of farnesylated PreA accumulated by ZmpSte24 inhibition are rapidly processed upon enzyme activation, yet abnormal nuclear phenotypes resulting from this accumulated PreA are slower to resolve. Recovery occurs more rapidly in proliferative cells, yet too quickly to require nuclear envelope breakdown, suggesting a structural reorganization of the lamina may constantly occur in cycling cells. We observed a much slower recovery of endogenous LaA maturation following washout of FTI or Lov than for Lop. This may not be surprising as our readout of LaA maturation requires only ZmpSte24 cleavage for Lop inhibition, whereas farnesylation, aaXing, carboxylmethylation and then ZmpSte24 cleavage all must occur following FTI or Lov treatment. Furthermore, in the case of ZmpSte24 there are no known substrates other than LaA, but there are ∼30–100 other substrates to compete for limited farnesylation, aaXing and carboxylmethylation enzymes.

These observations suggest that components of the nuclear lamina are readily accessible to processing enzymes such as the soluble farnesyltransferase and the integral membrane ZmpSte24, Rce1 and ICMT. Thus, the lamina may in fact be less static than is generally envisaged. Previous studies utilizing FRAP analyses have described GFP-LaA as essentially immobile [45], [46]. However, in addition to relying on exogenous lamins with large N-terminal fusion proteins, this technique only accounts for rather large-scale movement of lamins within the nucleus. If lamins do form structures similar to other intermediate filaments, then it is difficult to imagine how an entire population of PreA assembled into a static lamina could be readily accessible to the integral membrane processing enzymes such as ZmpSte24. If all of the C-terminal ‘tails’ were exposed on the surface of a lamin filament that was capable of dynamic ‘rolling’ and situated adjacent to the surface of the INM then this might permit sufficient association with the processing enzymes. Or there could be focal assembly and disassembly of lamin dimers that form the filaments, a model that has been proposed for cytoplasmic intermediate filaments [47]. However, this possibility would be hard to reconcile with the A-type lamin photobleaching data. An alternative explanation for the rapid processing of lamina-associated PreA by ZmpSte14 would be the lack of a filamentous lamina, at least in the conventional sense.. In the absence of direct observations on the organization of the somatic cell lamina, this latter suggestion cannot be entirely excluded.

The mechanism by which farnesylated PreA leads to HGPS or RD remains unknown. However, there is considerable evidence that a shift in the ratio of farnesylated A-type lamins is seriously detrimental [9], [26]. Previously, aberrant mitotic and post-mitotic aggregates of progerin have been described with a suggested role in the etiology of HGPS [42], [43]. We have observed identical results in ZmpSte24-null cells. Although the purpose of this study does not include investigating the mechanisms of PreA or progerin toxicity, our findings have added to the long list of associated phenotypes. We have found not only A-type lamins, emerin and LaB1 in these aggregates (Dechat et al., 2007), but also Sun2 and Nesprin 3, both LINC complex components. The displacement of these NE constituents from the newly forming post-mitotic NE is a possible mechanism by which aberrantly farnesylated lamins may be toxic. This could perturb the organization of nuclear structures such as chromatin and NPCs as well as the way in which the nucleus interacts with the cytoskeleton via the envelope spanning LINC-complexes.

A role for farnesylated PreA in acquired lipodystrophy in HIV patients receiving HAART treatment has not been conclusively proven. However, there is considerable circumstantial evidence that inhibition of ZmpSte24 by certain PIs may indeed contribute to this lipodystophy [15], [16]. And although it is unclear how PreA may induce lipodystrophy, expression of progerin has been reported to inhibit adipogenesis in human mesenchymal stem cells [48].

In the case of nonfarnesylated-PreA and HDJ-2, maturation following FTI washout in Saos-2 cells is considerably slower than is found for Lov washout. This discrepancy may result from both reduced clearance of the FTI and the geranylgeranylation of LaA [44]. In support of PreA geranylgeranylation, we have observed that GGTI must be utilized with the FTI to quantitatively inhibit maturation of LaA. When the rapidly diving Saos-2 cells were treated combinatorially with Lop, FTI and GGTI, the prenylation inhibitors blocked the aberrant cellular phenotype associated with Lop treatment-alone. However, upon washout of the FTI and GGTI the cells rapidly acquired the nuclear abnormalities and cytoplasmic aggregates that are normally observed following Lop treatment. We attribute this to the shift of accumulated nonprenylated PreA within the nuclear lamina to a toxic prenylated state that rapidly induces an aberrant cellular phenotype. Recently Lee et al. have reported that in cells lacking the β-subunit of farnesyl transferase there is a significant accumulation of PreA which is described as nonfarnesylated [49]. However, the apparent presence of mature LaA in these cells also suggests geranylgeranylation or some alternative mechanism is promoting lamin A processing. Another recent study addressing the subcellular localization of LaA processing described the relatively rapid recovery of mature LaA following FTI washout in the presence of cyclohexamide [50]. The discrepancy with our rather slower recovery (1.5h versus 4–5h with cyclohexamide) may be a reflection of the fact that these cells were expressing exogenous GFP-tagged lamins that appear localized in nucleoplasmic aggregates during FTI treatment. It is conceivable that this altered localization might enhance the exposure of PreA to the various lamin processing enzymes.

Although HGPS cells required 3 days to reacquire their aberrant nuclear profiles and cytoplasmic lamin aggregates after FTI washout, we suspect that this delayed recovery likely results from the extremely limited proliferative potential of these cells. Additionally, we cannot rule out that there may be different levels or activities of processing enzymes in these cells. Thus, it may be useful to determine the reversibility of lonafarnib, the FTI currently used in a clinical trial for HGPS, in order to evaluate any potential risks associated with skipped doses or rapid termination of the treatment in HGPS patients. Furthermore, the rate of PreA and progerin geranylgeranylation during lonafarnib treatment should be examined as this modification can delay proteolytic maturation of wild type PreA [51] which in turn could cause unintended accumulation of toxic PreA and progerin. We did observed that at doses sufficient to block PreA processing both Lov and the combinatorial FTI/GGTI treatment led to an obvious inhibition of cell growth over 48 hrs in Saos-2 cells. Thus, effective inhibition of progerin prenylation may require inhibitor concentrations too toxic for clinical use.

## Supporting Information

Figure S1 Inhibition of protein synthesis does not impair PreA processing following PI washout. Saos-2 cells were treated with cyclohexamide at the time of Lop washout. PreA levels dramatically disappear by 3 hrs following washout. Percent of mature LaA listed below.
(0.08 MB TIF)

Figure S2 Sun-2 and Nesprin-3 are present in abnormal cytoplasmic aggregates during mitosis and early G1 during Lop treatment. A 48hr treatment of Saos-2 cells with Lop led to the accumulation of Sun 2 and Nesprin-3 at LaA/C immunoreactive cytoplasmic aggregates in metaphase (upper panels) and early G1 (lower panels). DNA is labeled by Hoechst dye in blue. Bar, 10µm.
(0.46 MB TIF)

Figure S3 Multiple FTIs failed to permit rapid processivity of PreA following washout. As detected by anti-LaA immunoblots of Saos-2 cell lysates, PreA was refractory to processing following washout of 10µm L-744, 832 or 1µm BMS-214662. Percent of mature LaA listed below.
(0.79 MB TIF)

Figure S4 In HGPS cells, HDJ-2 exhibits prolonged maturation following FTI washout. (A) An anti-HA immunoblot of extracts from WT human fibroblasts expressing exogenous HA-progerin were either treated with DMSO (control) or FTI-277 for 48 hrs immediately following transfection. The FTI-277 treated progerin migrates more slowly. (B) In HGPS cells treated with FTI-277 for 96hrs, HDJ-2 is incompletely processed by 24hrs following FTI-277. Percent of mature HDJ-2 listed above.
(0.10 MB TIF)
