# Supplementary figures and images for: Dynamics of Lamin-A Processing Following Precursor Accumulation
Source: PLoS One. 2010 May 28;5(5):e10874. doi: 10.1371/journal.pone.0010874 (PMC2878336; doi:10.1371/journal.pone.0010874)

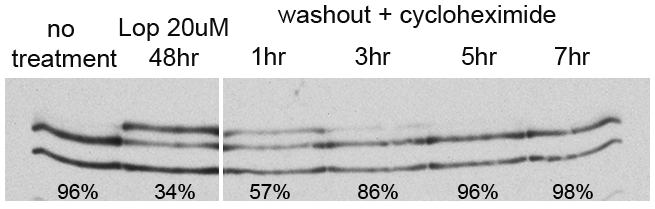

Supplement: Figure S1 — Inhibition of protein synthesis does not impair PreA processing following PI washout. Saos-2 cells were treated with cyclohexamide at the time of Lop washout. PreA levels dramatically disappear by 3 hrs following washout. Percent of mature LaA listed below. (0.08 MB TIF) [file pone.0010874.s001.tif]

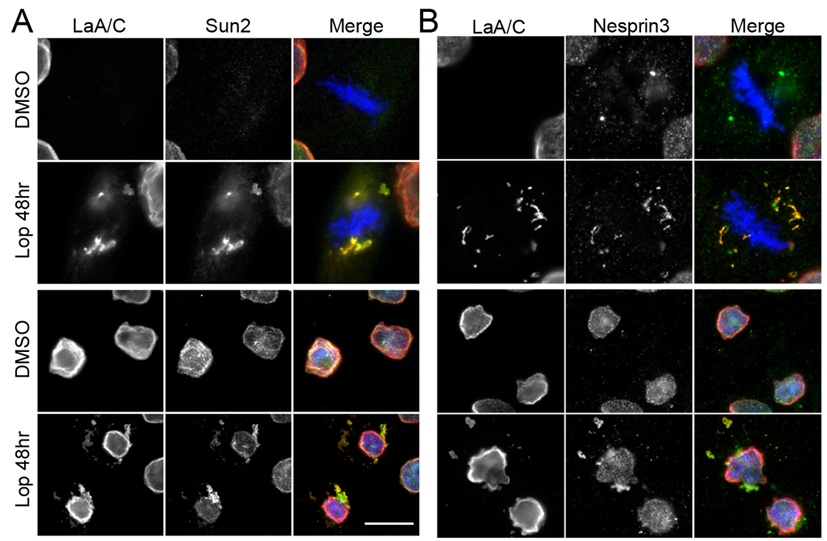

Supplement: Figure S2 — Sun-2 and Nesprin-3 are present in abnormal cytoplasmic aggregates during mitosis and early G1 during Lop treatment. A 48hr treatment of Saos-2 cells with Lop led to the accumulation of Sun 2 and Nesprin-3 at LaA/C immunoreactive cytoplasmic aggregates in metaphase (upper panels) and early G1 (lower panels). DNA is labeled by Hoechst dye in blue. Bar, 10µm. (0.46 MB TIF) [file pone.0010874.s002.tif]

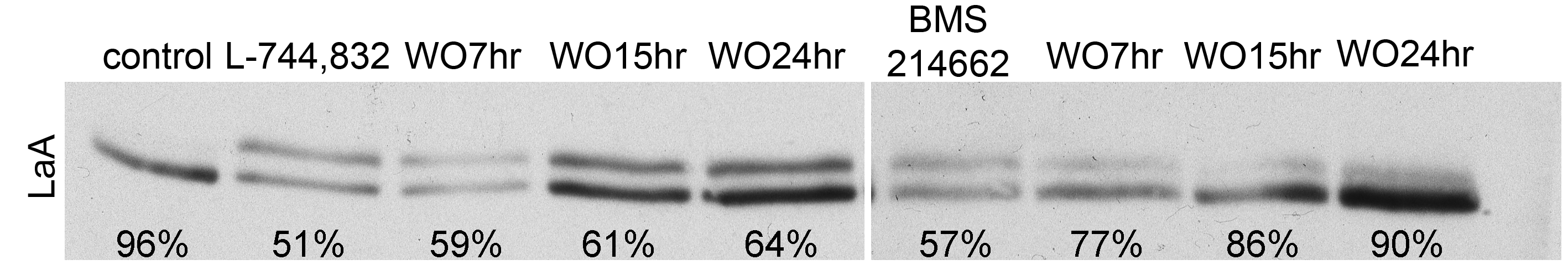

Supplement: Figure S3 — Multiple FTIs failed to permit rapid processivity of PreA following washout. As detected by anti-LaA immunoblots of Saos-2 cell lysates, PreA was refractory to processing following washout of 10µm L-744, 832 or 1µm BMS-214662. Percent of mature LaA listed below. (0.79 MB TIF) [file pone.0010874.s003.tif]

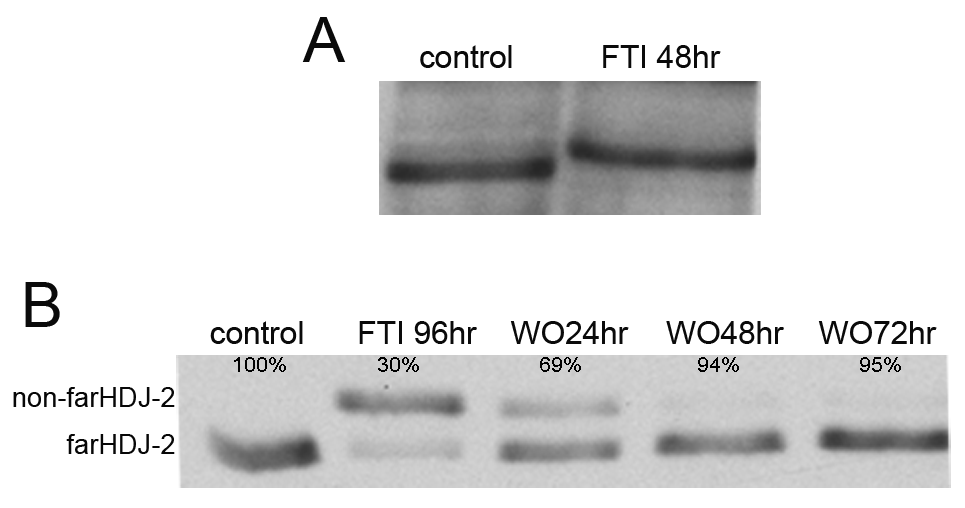

Supplement: Figure S4 — In HGPS cells, HDJ-2 exhibits prolonged maturation following FTI washout. (A) An anti-HA immunoblot of extracts from WT human fibroblasts expressing exogenous HA-progerin were either treated with DMSO (control) or FTI-277 for 48 hrs immediately following transfection. The FTI-277 treated progerin migrates more slowly. (B) In HGPS cells treated with FTI-277 for 96hrs, HDJ-2 is incompletely processed by 24hrs following FTI-277. Percent of mature HDJ-2 listed above. (0.10 MB TIF) [file pone.0010874.s004.tif]
